# The Relationship between Obligatory Exercise and Eating Attitudes, and the Mediating Role of Sociocultural Attitudes towards Appearance during the COVID-19 Pandemic

**DOI:** 10.3390/nu13124286

**Published:** 2021-11-27

**Authors:** Hongying Fan, Youteng Gan, Ruohang Wang, Siming Chen, Małgorzata Lipowska, Jianye Li, Keqiang Li, Daniel Krokosz, Yin Yang, Mariusz Lipowski

**Affiliations:** 1 School of Psychology, Beijing Sport University, Beijing 100084, China; 2020210226@bsu.edu.cn (Y.G.); wangruohang@bsu.edu.cn (R.W.); 13146930900@163.com (S.C.); yangyin@bsu.edu.cn (Y.Y.); 2 Institute of Psychology, University of Gdańsk, 80-309 Gdańsk, Poland; malgorzata.lipowska@ug.edu.pl; 3 Faculty of Physical Culture, Gdańsk University of Physical Education and Sport, Górskiego 1, 80-336 Gdańsk, Poland; jianye.li@awf.gda.pl (J.L.); keqiang.li@awf.gda.pl (K.L.); daniel.krokosz@awf.gda.pl (D.K.)

**Keywords:** eating habits, obligatory run, body esteem, SARS-CoV-2

## Abstract

A strong sociocultural context could affect an individual’s aesthetic standards. In order to achieve a socially recognized ideal appearance, obligatory exercisers might increase dieting behavior when exercise actions are disturbed, thereby placing the individual at risk of eating disorders. The current study mainly examined the relationship between obligatory exercise and eating attitudes during the COVID-19 pandemic, and considered the mediating role of externalized sociocultural attitudes towards appearance between the two. A total of 342 participants (175 females, 167 males) from various regions of China were invited to fill out the questionnaires including the Obligatory Exercise Questionnaire, the Sociocultural Attitudes Toward Appearance Questionnaire-3, and the Eating Attitudes Test. In total, 51.5% of the participants presented symptoms of an obligatory exercise behavior. Among them, males, young adults, and the participants with lower BMI had higher OEQ scores, whereas females and young adults had higher EAT-26 scores. Meanwhile, 9.4% of the participants might have had an eating disorder. The OEQ score was positively correlated with the EAT-26 total score as well as SATAQ-3 ‘Pressures’ and ‘Information’ subscales. In addition, the EAT-26 total score was positively correlated with the SATAQ-3 ‘Pressures’ and ‘Information’ subscales. Externalized sociocultural attitudes towards appearance served as a mediator between obligatory exercise behavior and eating attitudes, and the mediation effect accounted for 56.82% of the total effect. Obligatory exercise behavior may have an indirect effect on eating attitudes through sociocultural attitudes towards appearance. Given the sociocultural information and pressures, in order to maintain or pursue an ideal appearance, many people tend to keep a pathological diet. Thus, forming a positive and healthy social aesthetic orientation is beneficial in helping obligatory exercisers to develop reasonable eating habits.

## 1. Introduction

Physical activity (PA) is well-known for its beneficial effects for cognition [1,2]; however, it not only has the function of promoting personal physical health but can also reduce health and become an economic burden on society [3,4]. Moreover, exercise improves adverse symptoms in patients with depression and sleep disorders [5,6], promotes mental well-being (including emotional state and self-esteem), supports coping with alcohol problems [7], and increases positive emotions [8,9]. Previous research revealed that the life satisfaction of students who participated in extracurricular exercises was significantly higher in comparison to non-participating peers, indicating that exercise also improves life satisfaction [10]. However, in cases when exercise behavior is excessive, and people perceive it as the key life activity, ignoring their current physical condition or the social consequences of being obsessed with exercising (e.g., neglecting household chores, relationships, and obligations), such a behavior is called ‘obligatory exercise’ [11]. People presenting symptoms of the obligatory exercise behavior (so-called ‘obligatory exercisers’) strictly abide by their exercise plan, are over-engaged in it, and plan daily life around it [12,13]. As indicated by Dittmer et al. [14], they continue physical activity regardless of pain, often with obsession and other comorbid psychopathological behaviors. 

At present, obligatory exercise behavior is receiving considerable attention. For instance, it was found that exercise in weight management was associated with obligatory exercise in female college students [15]. Moreover, the reasons for obligatory exercise behaviors differ between men and women. Women mostly engage in obligatory exercises to enhance mood, tone, and health, while men engage in obligatory exercise to enhance tone, enjoyment, and physical attractiveness [16]. Of note, previous research has shown that obligatory exercisers generally have lower self-esteem and experience greater body dissatisfaction [17].

It is worth noting that obligatory exercise is also related to eating attitudes to some extent [18]. Some researchers and practitioners believe that if there is no disorder of eating attitude, obligatory exercise is rarely a problem. They indicate that people with negative attitudes are more likely to switch to exercise when they feel particularly distressed, as this reduces their emotional state [19]. In terms of factors having a potential influence on eating attitudes, Erol et al. [20] found that the body mass index (BMI) of men was not related to their eating attitudes and eating behaviors. However, compared with men, women’s eating attitudes and eating behaviors were strongly related to their BMI index [21]. Furthermore, adolescent girls and young women were more likely to suffer from eating disorders, with college women having the highest rate of eating disorders [22,23]. Furthermore, studies have shown that with an increase in work stress and BMI, the risk of eating disorders increases [24].

In general, people with negative eating behaviors face a high risk of eating disorders, which are characterized by abnormal eating behavior and psychopathology and are accompanied by a group of syndromes that include changes in body weight or physiological disorders, including anorexia nervosa, bulimia, and atypical eating disorders [25]. Notably, eating disorder patients tend to over-exercise in terms of exercise amount, frequency, or both [26]. In a study of 61 female casual triathletes, Elbourne and Chen [27] found a significant positive correlation between obligatory exercise and the prevalence of eating disorders. Although the research on the relationship between obligatory exercise and eating attitude is relatively abundant, Gapin and Petruzzello [28] believe that more research is needed to clarify this relationship and determine mediating or moderating factors.

Strong sociocultural context may affect an individual’s aesthetic standards, and the degree of personal recognition of this aesthetic may also affect behavior. Studies have shown that Hong Kong girls prefer slim bodies compared to Native American, white, and Hispanic girls [29], and Asian girls have a greater fear of fat [21,30,31]. In Western culture, mainstream media (such as TV shows, movies, and magazines) have shaped such an aesthetic concept, portraying the ideal female image as slim and young with a slender waist and fair skin [21,32,33]. Moreover, the media often promotes physical exercise as a mean of achieving slimness and gaining strength and even strongly suggests that anyone who is willing to spend time and energy to exercise can achieve the ideal figure. However, the history of exposure to the media predicts young people’s body dissatisfaction [34,35]. Moreover, the exercises for the management of appearance and weight are related to obligatory exercise behavior [15]. A similar discovery was made by Homan [36], who found that if women do not achieve their ideal body shape, they may feel dissatisfied with their bodies and feel ashamed or guilty for not achieving their exercise goals. Polivy and Herman [37] believe that the stress of achieving a slim female figure is an important risk factor that leads to disordered eating and obligatory exercise behaviors. In order to achieve the socially accepted ideal appearance, obligatory exercisers may increase their dieting behavior when exercise behavior is disturbed and further change their eating attitude. Therefore, it is worth exploring the role of the aesthetic standards that are shaped by the sociocultural context, in the relationship between obligatory exercise and eating attitudes. 

In the meantime, Cahill and Mussap [38] found that the sociocultural pressure is positively correlated with obligatory exercise among adult men and women and that the aesthetics shaped by the social culture may also have an impact on eating attitudes. For instance, research [39,40] suggests that the cultural pressure of women to lose weight and diet is related to severe eating disorders, and Toro et al. [41] found that the popular cultural aesthetic body model (slenderness) plays a leading role in eating disorders. Consequently, the media, as a sociocultural output medium, is often blamed as the perpetrator of bad eating attitudes [42]. Existing studies support the hypothesis of the close relation between obligatory exercise, sociocultural attitudes towards appearance, and eating attitudes.

However, in the current state of knowledge of the relationship between the abovementioned three factors, the aspect of sociocultural attitudes towards appearance has mainly been explored as the aspect of internalization of participants. This internalization involves the affirmation of socially defined ideals and taking action to realize these ideals, such as the thin-ideal and athletic-ideal, which can also be understood as the degree of personal desire to have a good body shape. Among them, the thin-ideal is believed to directly promote physical dissatisfaction and dieting, and these two variables increase the risk of eating pathology [43,44]. Homan [36] conducted a longitudinal study and found that athletic-ideal can predict changes in obligatory exercise but not body dissatisfaction or dieting, while internalization of the thin-ideal can predict changes in these three variables. Similarly, Bell et al. [45] found that the internalization of the athletic-ideal is not associated with body dissatisfaction, but it can directly predict dieting, bulimic symptoms, and obligatory exercise behavior. In brief, there are few studies discussing sociocultural attitudes towards appearance from an externalization perspective. Unlike internalization, externalization emphasizes the individual’s perception of aesthetic information and the pressure created by the media, i.e., the degree of recognition of these aesthetics. There is a clear gap in knowledge as to whether a tendency towards obligatory exercise affects one’s degree of recognition of the aesthetics shaped by the externalized sociocultural context and whether this degree of recognition affects one’s eating attitudes. 

In addition, the coronavirus disease pandemic (COVID-19) has seriously disturbed the global rhythm. Due to the absence of prophylactic vaccines, the effective strategies to mitigate the COVID-19 pandemic are mainly to limit the scope of people’s activities [46]. Italy, for example, imposed a blockade of the whole country in March 2020, during which all non-essential venues (such as bars and restaurants) were closed, and people were required to not leave their houses unless it was proved necessary, otherwise meeting severe sanctions. The Chinese government has implemented strict social distancing measures to curb the spread of COVID-19, such as shutting down non-essential businesses and public transport. Nevertheless, this popular measure not only brings great challenges and changes to people’s daily life and social style but also has significant effects on people’s physical and mental health [47]. Although the COVID-19 pandemic is a rare event, its impact may be printed on each individual concerned [48]. To a degree, Holmes et al. [49] believe that research on the health effects of the COVID-19 pandemic should be identified as a priority. Therefore, this study intends to focus on the pandemic period while combining demographic information to explore the characteristics of people’s exercise behaviors, diet, social media stress, and information perception in the context of the epidemic.

In the light of the above, the main purpose of this research is to further explore the relationship between obligatory exercise, externalized sociocultural attitudes towards appearance, and eating attitudes during the COVID-19 pandemic. Based on the above discussion, our assumptions are as follows: (1) affected by the pandemic, people with different demographic characteristics vary in these three aspects (obligatory exercise, externalized sociocultural attitudes towards appearance, and eating attitudes); (2) there is a significant correlation between these three factors; (3) externalized sociocultural attitudes play a mediating role between obligatory exercise behavior and eating attitude.

## 2. Materials and Methods

### 2.1. Procedure

The data used for this study were part of a large international research project registered in the Protocol Registration and Results System (ClinicalTrials.gov; https://clinicaltrials.gov/ct2/show/NCT04432038). The procedure carried out in the project consisted of an online survey. Data in this study were collected between April 2020 and July 2021, in the period of the COVID-19 pandemic, from the Chinese population. The study was carried out in accordance with the Code of Ethics of the World Medical Association (Declaration of Helsinki) for research involving humans. The protocol of this study was approved by the Ethics Board for Research Projects at the Institute of Psychology, University of Gdańsk, Poland (decision no. 33/2020). All participants were acquainted with the purpose of the conducted research, and asked to complete an electronic informed consent form before registration on the project’s website.

According to the report on the COVID-19 epidemic situation in China, reported by the National Health Commission of the Republic of China, as of 24:00 (12.00 PM) on 31 July 2021, the total number of confirmed cases in 31 provinces (autonomous regions, municipalities directly under the Central Government) and Xinjiang Production and Construction Corps was 93,005.

### 2.2. Participants

A total of 342 participants (175 females and 167 males) from various regions of China were invited to fill out the study’s questionnaires. The following inclusion criteria were used: age 18+, Chinese nationality, residence in China, and no physical disability or somatic diseases that prevent physical activity. The criteria were verified with the help of questions on socio-demographics and health, which allowed for the determination of the exclusion factors. 

During the data collecting period, 632 respondents filled in the survey in Chinese. Due to errors in filling out the questionnaires (incompleteness of the obtained research data) and the participation of people of a nationality other than Chinese, 198 questionnaires were excluded from the study. Due to the existence of exclusion criteria, another 92 sets of results were not included in this study.

All included participants spoke Chinese as their native language. The age range was 18–72 years old (*M* = 30.46, *SD* = 12.55), of which 46.78% were ≤24 years old, 25.73% were 25–34 years old, 10.82% were 35–44 years old, 9.94% were 45–54 years old, and 6.73% were ≥55 years old. In addition, 17.84% of people lived in rural areas or township (up to 100,000 inhabitants), 20.17% in medium-sized cities (100,000–1,000,000 inhabitants), and 61.99% in large cities (more than 1 million inhabitants). The BMI range of the participants was 14.88–33.83 (*M* = 22.27, *SD* = 3.729), and 36 people fell into the ‘thin’ category (BMI ≤ 18.4), 206 were ‘normal’ (BMI was 18.5–23.9), 69 were ‘overweight’ (BMI was 24.0–27.9), and 29 were in the ‘obesity’ category (BMI ≥ 28.0). 

### 2.3. Methods

#### 2.3.1. The Obligatory Exercise Questionnaire

The Obligatory Exercise Questionnaire (OEQ) was used to measure obligatory exercise behavior [50]. The survey, which consists of 20 items, is scored on a 4-point Likert scale (1 = never, 2 = sometimes, 3 = usually, and 4 = always), and the higher the score, the stronger the sense of the obligatory exercise. In addition, a person with a total score equal to or higher than 50 may be considered an obligatory exerciser. Of note, Cronbach’s alpha was 0.86 for the OEQ in the present study. 

#### 2.3.2. The Eating Attitudes Test

The Eating Attitudes Test (EAT-26) was used to evaluate the cognitive, emotional, and behavioral tendencies in eating [51]. EAT-26 consists of 26 items measuring three factors: dieting, bulimia, food preoccupation (abbreviated as bulimia), and oral control. Items are scored on a Likert scale (never, rarely, or sometimes = 0, often = 1, usually = 2, always = 3, with item 26 reverse-coded). For the purpose of this study, we used the ‘screening scoring’ method to calculate scores, and the higher the result, the more serious the participant’s eating attitudes and behavior problems. A total score of 20 or more may indicate an eating disorder. Cronbach’s alpha was 0.73 for the EAT-26 in the present study.

#### 2.3.3. Sociocultural Attitudes towards Appearance Questionnaire-3

We used two subscales from the Sociocultural Attitudes Towards Appearance Questionnaire-3 (SATAQ-3), Pressures and Information, that measure the extent to which individuals use the unrealistic body image promoted by the media as a body standard under the influence of the sociocultural context [52]. All items are scored on a 5-point Likert scale, ranging from 1 = ‘definitely disagree’ to 5 = ‘definitely agree’. The Pressures subscale consists of seven items and is used to measure the degree of pressure which an individual perceives when coming across influential material in the media (such as ‘I have felt pressure from TV or magazines to lose weight’). The Information subscale consists of nine items and is used to measure the degree to which an individual recognizes the media as a source of fashion and attractiveness (such as ‘TV programs are an important source of information about fashion and being attractive’). These two subscales together emphasize the impact of the sociocultural context on the individual aesthetics, and the higher the scores, the greater the impact. Cronbach’s alpha was 0.843 for the SATAQ-3 in the present study.

#### 2.3.4. Data Analysis

Data analysis was performed using SPSS 26.0 (IBM, Armonk, New York, NY, USA) and AMOS 26.0 (IBM, Armonk, New York, NY, USA). T-test or one-way ANOVA (including pairwise comparison) was used to compare the differences in scores of participants regarding different demographic characteristics. Pearson’s correlation analysis was used to explore the relationship between the scores of OEQ, EAT-26 (dieting, bulimia, and oral control), and SATAQ (Pressures and Information). Multiple linear regression (stepwise method) was used, taking the demographic characteristics, OEQ, and SATAQ as predictors, to explore the relationships between these predictors and EAT-26. AMOS 26.0 was used to clarify the influence path of participants’ obligatory exercise behaviors and external sociocultural attitudes towards appearance on eating attitudes. Bootstrapping was used to test the mediating role of external sociocultural attitudes towards appearance between obligatory exercise behavior and eating attitudes. We used the *p*-value of 0.05 to estimate the significance of the differences.

## 3. Results

### 3.1. Participants’ Exercise Behavior, Eating Attitudes, and Degree of Sociocultural Influence

The differences between groups distinguished on the basis of the variation in demographic variables in the OEQ, EAT-26, and SATAQ are presented in Table 1. It can be found that, in case of OEQ scores, males, young adults (age ≤ 34), and those with BMI < 24.0 (thin or normal) received higher results, while, on the EAT-26 scale, females and young adults (age ≤ 34) received higher total scores. In the case of the SATAQ-3, females and people aged 24–35 received higher scores on both the Pressures and Information subscales, while those living in large cities and those having BMI ≥ 24 (overweight or obesity) received lower scores on the Pressures subscale. In addition, 176 (51.5%) and 32 (9.4%) participants reached the cut-off point for the obligatory exercise behavior and eating disorders, respectively.

### 3.2. The Relationship between OEQ, EAT-26, and SATAQ Scores

As shown in Table 2, Pearson’s correlation analysis showed that OEQ score was positively correlated with bulimia (*r* = 0.19, *p* < 0.01), oral control (*r* = 0.11, *p* < 0.05), EAT-26 total score (*r* = 0.11, *p* < 0.05), Pressures (*r* = 0.37, *p* < 0.01), and Information (*r* = 0.32, *p* < 0.01). In addition, the EAT-26 total score was positively correlated with Pressures *(r* = 0.17, *p* < 0.01) and Information (*r* = 0.20, *p* < 0.01).

### 3.3. Prediction Results of EAT-26 by Demographic Characteristics, OEQ, and SATAQ Scales

When considering the prediction of EAT-26 parameters by demographic characteristics, OEQ, and SATAQ scales, the adjusted R^2^ ranged from 0.013 to 0.124 in the participants. Specifically, gender predicted dieting and total score; age predicted bulimia and total score; BMI predicted oral control; Pressures predicted bulimia; and Information predicted oral control and total score (see Table 3).

### 3.4. Mediating Effect of Sociocultural Attitudes towards Appearance

**Figure 1:**
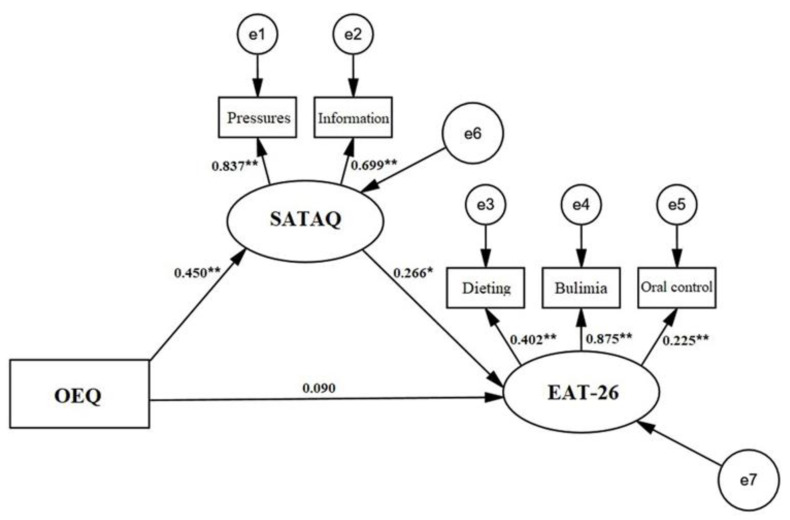
Path analysis of the relationship between OEQ, EAT-26, and SATAQ of participants. Note: OEQ = Obligatory Exercise Questionnaire; SATAQ = Sociocultural Attitudes Towards Appearance Questionnaire; EAT-26 = Eating Attitudes Test-26; * *p* < 0.05, ** *p* < 0.01.

The structural equation model (SEM) with OEQ as the independent variable, EAT-26 as the dependent variable, and SATAQ as the mediating variable (see Figure 1) was found to be well-fitted (*χ*^2^/df = 2.360, AGFI = 0.954, CFI = 0.966, GFI = 0.985, IFI = 0.967, RMSEA = 0.0469, SRMR = 0.063, TLI = 0.928). The standardized path coefficients showed that OEQ predicts SATAQ-3’s scores (*β* = 0.450, *p* < 0.01) and that SATAQ-3 predicts EAT-26’s scores (*β* = 0.266, *p* < 0.05). However, there was no relationship between OEQ and EAT-26 (Figure 1).

Furthermore, we used the deviation-corrected percentile bootstrap method to extract 2000 bootstrap samples to test the significance of the mediation effect. The results showed that the confidence intervals of the mediating effect and the direct effects are (0.004, 0.059) (excluding 0) and (−0.013, 0.054) (including 0), which indicates that the mediating effect is significant, but the direct effect is insignificant. Therefore, externalized sociocultural attitudes towards appearance played a complete mediating role between obligatory exercise behavior and eating attitudes, and the mediation effect accounted for 56.82% of the total effect (see Table 4).

## 4. Discussion

The purpose of the current study was to examine the relationship between obligatory exercise, externalized sociocultural attitudes towards appearance (externalized sociocultural attitudes), and eating attitudes during the COVID-19 pandemic and to consider the mediating role of the externalized sociocultural attitudes between the obligatory exercise and eating attitudes. Our study supports the hypothesis of the positive correlation among these three factors, as well as our initial assumption that externalized sociocultural attitudes play a mediating role between obligatory exercise and eating attitudes. Moreover, we found significant differences in obligatory exercise, externalized sociocultural attitudes towards appearance and eating attitudes between people with different demographic characteristics.

Analyzing the differences in the OEQ results, we found that the score of the males was significantly higher than that of the females, which is similar to the results obtained by Tata et al. [53], who believe that these differences can probably be explained by sex differences in the perception of weight. Moreover, in our study, young people (≤24 years old) had the highest OEQ scores, and a study of 589 college students (mean age = 20) by Guidi et al. [54] found that young people may represent high-risk groups for compulsive exercise. Regarding the place of residence, we found that people living in medium-sized cities received the highest scores in OEQ, which may be due to stricter epidemic prevention and control measures in China’s agglomeration areas (thus, there may be more restrictions on exercise behavior) in comparison to rural or township areas where there are relatively large per capita activity areas (thus, less affected by the epidemic). We also found that people with BMI in the ‘thin’ and ‘normal’ range received significantly higher OEQ scores, and a reasonable explanation is that these groups tend to adhere to exercise behaviors in order to maintain or pursue a better body level. Nevertheless, in this area of research, some scholars believe that obligatory exercisers have lower BMIs than non-obligatory exercisers [55,56], but others believe that there is no difference in BMI between the two [57], and more detailed investigation can be considered in future research. 

In contrast to the OEQ results, we found that the total scores for the EAT-26 were higher among females than among males. From the epidemiological perspective, eating disorders are more common among women [58,59]. Moreover, the EAT-26 total score in the age group of ‘25–34’ was also significantly higher than that of other age groups. Many people at such a young age are still in the early stages of their career or have just stabilized at work, must cope with the pressures of marriage, a mortgage, or raising young children, or a combination thereof. Pressure is thought to affect eating behavior [60]: people tend to change their original eating habits to cope with stress [61,62,63,64]. Therefore, higher life stress may be the reason for the abnormal eating attitudes of these groups. In addition, this study did not find an association between the place of residence or BMI and eating attitudes. 

In the two external dimensions of SATAQ-3 (Pressure and Information), our study revealed that females feel more pressure from the media reports on appearances and have a higher degree of recognition of such information. Similarly, Turel et al. [65] found that females experience more appearance-related social stress than men to achieve an ideal body; moreover, males seem to be less targeted by the media, so they are under less strict pressure to lose weight [66]. In other words, female norms in this regard may be more restricted than male norms. In tandem, social media exposure more often affects female desire for cosmetic surgery [67,68], which also supports the thesis that females are more vulnerable to media information. Additionally, in our study, the age group ’25–34’ received the highest score on Pressure and Information, probably because people at this age have more purchasing power and are more likely to be targeted by the media than younger groups, while in the case of older people, dissatisfaction with appearance decreases with age [69,70], so it is reasonable that their scores are lower. Interestingly, we found that people living in large cities felt less media pressure than those from other types of places of residence, which can be explained by the fact that the larger and more urbanized a community is, the more pluralistic it is [71]. Pluralistic aesthetics are inclusive and can reduce the influence of the media to a certain extent. Moreover, we found that the participants with BMI < 24.0 scored higher in Pressure, reflecting their more rigorous approach to the body. In contrast, the research by Jeffers et al. [72] pointed at a positive correlation between BMI and media pressure; however, the authors concluded that this result may be related to the young sample in their study (mean age = 18.85).

In addition to demographic differences, we examined the interrelationships between these three main variables in our study, and we found that there is a very weak positive correlation between the obligatory exercise behaviors and eating attitudes, which is in line with some previous discoveries [27,73]. However, some scholars believe that in the absence of negative emotional motivation, obligatory exercisers may not be so worried about diet [19]. Thus, it was reasonable to speculate that there may be other variables that affect the relationship between the two. Of note, we found that the score of OEQ was positively correlated with the Pressure and Information subscales of the SATAQ-3. Although the causality of this relationship cannot be determined, our results revealed that people’s attitudes towards obligatory exercise behavior is indeed related to their recognition of the aesthetics created by the media. These observations are partially confirmed by earlier reports; e.g., Goodwin et al. [74] have found that sociocultural factors have a significant influence on the obligatory exercise of adolescent boys and girls, which further emphasizes the close relationship between these factors. Previous research also confirmed that sociocultural factors are associated with eating attitudes [75,76,77]. To a certain degree, the external pressure and information constantly instilling the concept of beauty culture may affect people’s eating attitudes and prompt them to achieve socially acceptable ‘beauty’ through behaviors such as dieting and compulsive eating. Hence, as the main force of cultural output, the media needs to bear more responsibility for creating a sociocultural aesthetic context.

Furthermore, when the demographic variables, OEQ, and SATAQ scales were used to predict EAT-26, we found that gender and age predicted different components of eating attitudes separately, while similar results were presented for Pressure and Information, consistent with our previous findings. Meanwhile, BMI negatively predicted oral control, which explained why the group with lower BMI was more strictly controlled on diet. Significantly, in the SEM, we found that obligatory exercise predicts externalized sociocultural attitudes towards appearance and that these attitudes predict eating attitudes. In other words, our study revealed that the more intense the sense of obligation to exercise is, the more people recognize the aesthetics shaped by the sociocultural context, and the higher the degree of such a recognition, the more extreme the eating attitude is. Of course, this is also related to the spurious aesthetic culture created by today’s media. Moreover, as expected, we found a third variable that well explains the relationship between obligatory exercise behavior and eating attitude. In our mediation model, obligatory exercise behavior did not directly affect eating attitudes but did indirectly affect them—through the mediating effect of externalized sociocultural attitudes towards appearance. It is worth noting that some scholars have found that there is a positive correlation between appearance comparison and obligatory exercise [78], and a metanalysis conducted by Alcaraz-Ibáñez et al. [79] pointed out that body dissatisfaction may be one of the underlying causes of morbid exercise behavior (MEB), which indicates that groups with a tendency towards obligatory exercise behavior are more likely to be dissatisfied with their bodies. As an effective way to improve or maintain the appearance of the body, exercise behavior itself is associated with creating a satisfactory appearance. Therefore, when the aesthetic concept promoted by the media is consistent with the ideal figure that the exercisers expect, they will agree more with such messages and regard them as the standard for their own exercise behavior. However, it may also make their minds unwittingly occupied by the media information and put them in a position where they must bear the appearance-related sociocultural pressure brought by the media. In general, if the idea spread by the media is healthy and positive, these problems may not arise. Unfortunately, the reality differs from this wishful picture. In the context of modern culture, the Chinese rather than Western media has placed more emphasis on female body image, and young Chinese women are more susceptible to pressure from Chinese and Asian mass media [80]. Hong Kong’s mass media, for instance, emphasizes the ideal of thin-beauty and presents this aesthetic view through eye-catching methods such as ultra-thin models, movie stars, and pop singers [81]. Worth noting is a study of Chinese ‘rabbit’ groups (derived from the Chinese homonym of ‘rabbit’ and ‘Spit’), which found that most ‘rabbits’ have strict physical discipline, but under the misleading context that society creates where ‘thin is ideal’, the ‘rabbit’ equates thinness with success, regards building a perfect body as a shortcut to happiness, and thus often suffers from eating disorders in this drastic process of weight loss. Therefore, Dong [82] believes that it is worth considering how to formulate a reasonable orientation of aesthetic culture. Zhang [83] found that some Chinese women are worried about the superficial and extreme standards of beauty advocated by the media. The so-called ‘A4 waist challenge’ and ‘cartoon waist challenge’, recently launched by Chinese social media, aroused doubt and criticism from many people. Jackson et al. [84] revealed that among women who were exposed to A4 challenge images, body trait dissatisfaction predicted a post-exposure state of dissatisfaction. Of note, affected by the COVID-19 pandemic, young adults showed a greater increase in social media use [85], and people have increased usage of digital media near bedtime [86]. This suggests that the demand for social distancing could increase the use of social media as a means of communication [87] and access to real-time information. Unfortunately, however, as far as we know, the use of mobile phone applications and microblogs is positively correlated with eating disorders [88]. In brief, research shows that there are many deviations in the dissemination of aesthetic culture by the media, and these deviations seriously affect people’s attitude towards eating [89]. In addition, a longitudinal study from Spain also found that adolescents’ body satisfaction and perceptions of body image are potential predictors of increased eating disorders, and sociocultural factors are correlated with body satisfaction and body image [90]. Consequently, it can be concluded that unrealistic appearance-related sociocultural information and strong pressures are the risk factors for eating disorders. Although attending exercise is beneficial to health, it may also negatively influence people’s perception of health goals [91], and to some extent, influence them to have a stronger sense of identity with the culture promoted by social media, thus leading them to a cognitive bias about the aesthetic perception of stature under the influence of media, and this comprehension bias raises more problems about eating attitudes.

For exercisers, because their attitudes towards obligatory exercise were closely related to their recognition of the media’s aesthetics, this study holds that in order to avoid the extreme eating attitude of this group, we should create a healthy aesthetic culture context. That is to say, the policymakers need to take effective measures to control and correct the media’s unrealistic description of the so-called idealized body in order to minimize the risk of eating disorders for obligatory exercisers to the greatest extent. In short, given the sociocultural Information and Pressure, in order to maintain or pursue an ideal appearance, many people tend to keep a pathological diet. Thus, creating a positive and healthy social aesthetic orientation is beneficial to helping obligatory exercisers develop reasonable eating habits. Moreover, due to the impact of the COVID-19 pandemic, people may increase the frequency of using online software during the reduction of social activities, which also suggests that the relevant departments should supervise the content presented in the media. Meanwhile, the current study also put forward an idea of whether media schools could incorporate some knowledge about psychological and psychopathological aspects into the curriculum system, or hold regular lectures on similar topics, when developing back-up talents in the field. More broadly, it might be possible to radically reduce the production of similar behaviors, only if people in the future of the media become more clearly informed of how their way of advocacy will affect society.

## 5. Limitations

There are some limitations of this study: First, only externalized sociocultural attitudes were considered as a mediating variable, and their index is relatively simple. Future research can consider introducing more intermediary or regulatory variables, such as physical satisfaction, to further improve the construction of the model in this study. Second, this study was a cross-sectional study, which can only indicate correlations between obligatory exercise, externalized sociocultural attitudes towards appearance, and eating attitudes but cannot reveal the causality among them. Future research should thus implement a longitudinal or experimental design, or both, in order to deepen the understanding of the problem raised in the current study. Finally, when classifying the population in this study, some categories contained relatively small numbers of people, which may have some impact on the results, and future research could pay more attention and achieve more balanced population proportions.

## 6. Conclusions

To summarize, the present study found that there is a correlation between obligatory exercise, externalized sociocultural attitudes towards appearance, and eating attitudes, and externalized sociocultural attitudes play a completely intermediary role between obligatory exercise and eating attitudes. This shows that people’s attitude towards obligatory exercise affects their recognition of the aesthetics formed by sociocultural context, which in turn affects their attitude towards eating. Social media currently promotes a negative aesthetic culture, which may lead to more exercisers developing morbid eating behavior. Therefore, the results of this study may provide insights into how to help people develop a healthy eating concept. Meanwhile, it is our hope that this research will draw attention to the increasingly abnormal aesthetics conveyed in sociocultural attitudes and reduce the risk of eating disorders during the COVID-19 epidemic.

## Figures and Tables

**Table 1:** Descriptive statistics and differential analysis of participants’ scores on the OEQ, EAT-26, and SATAQ scales.

Variables	Category (*N*)	OEQ	EAT-26				SATAQ	
			Dieting	Bulimia	Oral Control	Total Score	Pressures	Information
Gender	Female (175)	45.80 ± 10.159	6.83 ± 5.371	1.06 ± 1.708	2.30 ± 2.525	10.19 ± 6.775	20.50 ± 6.961	26.98 ± 5.406
	Male (167)	51.21 ± 9.705	5.50 ± 5.212	1.04 ± 1.468	1.75 ± 2.133	8.26 ± 6.548	18.25 ± 6.840	24.81 ± 5.383
	t value	−5.031 ***	2.335 **	0.123	2.165 *	2.678 **	3.015 **	3.706 ***
Age	≤24 years (160)	51.09 ± 9.698 ^a^	5.64 ± 5.514 ^a^	1.18 ± 1.644	2.31 ± 2.403 ^a^	9.11 ± 6.744 ^ad^	19.34 ± 6.698 ^a^	25.15 ± 5.345 ^a^
	25~34 years (88)	50.00 ± 9.261 ^a^	8.55 ± 5.498 ^b^	1.23 ± 1.687	2.45 ± 2.382 ^a^	12.23 ± 6.959 ^b^	21.42 ± 6546 ^b^	28.55 ± 5.228 ^b^
	35~44 years (37)	40.65 ± 9.956 ^b^	4.19 ± 3.511 ^a^	0.73 ± 1.592	0.92 ± 1.422 ^b^	5.84 ± 4.947 ^c^	16.97 ± 7.392 ^a^	24.35 ± 5.427 ^a^
	45~54 years (34)	44.12 ± 8.693 ^b^	5.26 ± 4.882 ^a^	0.50 ± 1.052	1.79 ± 2.750 ^ab^	7.56 ± 6.325 ^cd^	18.18 ± 8.204 ^a^	25.28 ± 5.484 ^a^
	≥55 years (23)	43.00 ± 11.107 ^b^	5.43 ± 3.449 ^a^	0.74 ± 1.322	0.70 ± 1.329 ^b^	6.87 ± 3.900 ^cd^	17.83 ± 6.386 ^a^	24.74 ± 4.683 ^a^
	F value	13.228 ***	6.832 ***	2.180	5.540 ***	8.688 ***	3.622 **	7.477 ***
Residence	Rural or township (61)	48.75 ± 11.067 ^ab^	5.20 ± 5.102	1.33 ± 1.895	1.95 ± 2.617	8.48 ± 7.108	20.82 ± 7.284 ^a^	26.41 ± 5.719
	Medium-sized city (69)	51.65 ± 10.054 ^a^	6.30 ± 5.877	1.14 ± 1.683	2.19 ± 2.088	9.64 ± 6.575	20.64 ± 6.642 ^a^	26.17 ± 6.007
	Large city (212)	47.31 ± 9.947 ^b^	6.42 ± 5.197	0.93 ± 1.459	2.01 ± 2.365	9.35 ± 6.675	18.59 ± 6.913 ^b^	25.70 ± 5.265
	F value	4.782 **	1.283	1.618	0.197	0.541	3.816 *	0.487
Body Mass Index BMI *	≤18.4 Thin (36)	50.39 ± 13.096 ^a^	4.36 ± 5.452	1.06 ± 1.511	3.78 ± 3.415 ^a^	9.19 ± 6.899	21.29 ± 7.436 ^a^	26.28 ± 5.348 ^ab^
	18.5~23.9 Average (206)	50.21 ± 9.729 ^a^	6.66 ± 5.589	1.12 ± 1.559	2.12 ± 2.200 ^b^	9.88 ± 6.805	20.49 ± 6.625 ^a^	26.59 ± 5.589 ^a^
	24.0~27.9 Overweight (69)	44.42 ± 8.740 ^b^	5.70 ± 4.769	0.88 ± 1.605	1.23 ± 1.783 ^c^	7.81 ± 6.603	16.33 ± 6.917 ^b^	24.38 ± 5.056 ^b^
	≥28 Obesity (29)	42.48 ± 9.206 ^b^	6.34 ± 4.228	0.93 ± 1.963	1.03 ± 1.500 ^c^	8.31 ± 5.971	16.45 ± 6.384 ^b^	24.21 ± 4.924 ^b^
	F value	9.933 ***	2.155	0.437	12.295 ***	1.851	9.288 ***	3.958 **

Note: Independent-samples t-tests were used for analyses by gender, and one-way analysis of variance tests (ANOVA) was used for analyses by age, residence, or BMI. ^abcd^ the same letter means that there is no difference between pairwise comparisons within the group, and different letters mean that there is a difference. * *p* < 0.05, ** *p* < 0.01, *** *p* < 0.001.

**Table 2:** Pearson correlation between OEQ, EAT-26, and SATAQ scores.

	1	2	3	4	5	6	7
OEQ	1						
EAT-26 Dieting	0.035	1					
EAT-26 Bulimia	0.185 **	0.357 **	1				
EAT-26 Oral control	0.111 *	0.044	0.192 **	1			
EAT-26 Total score	0.111 *	0.894 **	0.588 **	0.431 **	1		
SATAQ Pressures	0.372 **	0.071	0.233 **	0.161 **	0.168 **	1	
SATAQ Information	0.324 **	0.124 *	0.150 **	0.199 **	0.204 **	0.585 **	1

Note: * *p* < 0.05, ** *p* < 0.01.

**Table 3:** Stepwise multiple linear regression predicting EAT-26 by demographic characteristics, OEQ and SATAQ scales.

	Adjusted R^2^	β (B, Standard Error) Predictors
EAT-26		
Dieting	0.013	−0.128 (−1.362, 0.576) Gender *
Bulimia	0.061	0.220 (0.050, 0.012) SATAQ Pressures **
		−0.110 (0.014, 0.007) Age *
Oral control	0.124	−0.311 (−0.196, 0.032) BMI **
		0.133 (0.057, 0.022) SATAQ Information *
Total score	0.070	0.164 (0.202, 0.066) SATAQ Information **
		−0.164 (−0.088, 0.028) Age **
		−0.127 (−1.706, 0.723) Gender *

Note: Gender was dummy coded such that 1 = male and 2 = female; Bs were unstandardized coefficients; * *p* < 0.05, ** *p* < 0.01.

**Table 4:** Bootstrap analysis of the mediation effect size and significance test of SATAQ in OEQ and EAT-26.

Path	Standardized Effect Size	Standard Error	Effect Size	95% CI	
	(Effect)	(Boot SE)	(%)	LL	UL
OEQ→EAT-26 (Direct effect)	0.090	0.017	42.86	(−0.013, 0.054)	
OEQ→SATAQ→EAT-26 (Mediation effect)	0.120 **	0.014	57.14	(0.004, 0.059)	
OEQ→EAT-26 (Total effect)	0.210 **	0.017	100	(0.016, 0.084)	

Note: CI confidence interval, LL lower limit, UL upper limit. ** *p* < 0.01.

## Data Availability

The data from this study are available from the corresponding author upon request.
